# Striated Muscle Evaluation Based on Velocity and Amortization Ratio of Mechanical Impulse Propagation in Simulated Microgravity Environment

**DOI:** 10.3390/biology11111677

**Published:** 2022-11-18

**Authors:** Alexandru Nistorescu, Stefan Sebastian Busnatu, Adrian Dinculescu, Gabriel Olteanu, Mihaela Marin, Cosmina Elena Jercalau, Cristian Vizitiu, Ioana Raluca Papacocea

**Affiliations:** 1 Space Applications for Human Health and Safety Department, Institute of Space Science, 077125 Măgurele, Romania; 2 Department of Cardiology, University of Medicine and Pharmacy “Carol Davila”, Emergency Hospital “Bagdasar-Arseni”, 050474 Bucharest, Romania; 3 Department of Automatics and Information Technology, Faculty of Electrical Engineering and Computer Science, Transilvania University of Brasov, 500024 Brasov, Romania; 4 Physiology Department, Carol Davila University of Medicine and Pharmacy, 050474 Bucharest, Romania

**Keywords:** muscle mechanical properties, dry immersion, simulated microgravity environment, striated muscle, mechanical excitation, imponderability, muscle loss

## Abstract

**Simple Summary:**

Long-duration space flight missions impose extreme physiological stress and changes on the crew due to the microgravity exposure. The herein study was performed by using MusTone, a device developed by the Institute of Space Science, to understand the influence of microgravity physiological stress on striated muscles and to provide countermeasures that are able to minimize the negative effects of weightlessness on musculoskeletal function. The results emerged following a statistical analysis performed on the data collected from six subjects engaged in a 21-days Dry Immersion (DI) experiment. Two parameters of muscles’ fibers behavior in the longitudinal direction were extracted and analyzed (i.e., propagation velocity and amortization ratio). It was confirmed that muscle deconditioning is time-dependent and also that, as muscle exposure to Dry Immersion increases, the amortization ratio increases and is more significant in the distal position. Moreover, muscle deconditioning occurs in a gradient: it starts first in distal points and affects more the distal muscles, those which are involved in posture maintenance and have an antigravity role. The results are important when taking into account space tourism developments, as long as astronauts and space tourism candidates will require a reliable real-time and non-invasive method able to quantify the muscle dynamic changes.

**Abstract:**

Long-duration space flight missions impose extreme physiological stress and/or changes, such as musculoskeletal function degradation, on the crew due to the microgravity exposure. A great deal of research studies have been conducted in order to understand these physiological stress influences and to provide countermeasures to minimize the observed negative effects of weightlessness exposure on musculoskeletal function. Among others, studies and experiments have been conducted in DI analogue Earth-based facilities in order to reproduce the weightlessness negative effects on the human body. This paper presents a complex muscular analysis of mechanical wave propagation in striated muscle, using MusTone, a device developed in-house at the Institute of Space Science, Romania. The data were collected during a 21-day DI campaign in order to investigate muscle fibers’ behavior in longitudinal direction, after applying a mechanical impulse, taking into account two particular parameters, namely propagation velocity and amortization ratio. The parameters were determined based on the wave-propagation data collected from five points (one impact point, two distal direction points, and two proximal direction points) along the muscle fiber. By statistically analyzing propagation velocity and amortization ratio parameters, the study revealed that muscle deconditioning is time dependent, the amortization ratio is more significant in the distal direction, and the lower fibers are affected the most.

## 1. Introduction

Since the first ever 89 min human exposure to microgravity on 12 April 1961, up until the current regular long-duration missions (LDMs) in microgravity, numerous studies have highlighted physiological and biomechanical human body adaptation during space explorations. In this respect, given the absence of the static loading and drastic limitations in dynamic loading specific to 1-g, the human musculoskeletal system is led toward hypofunction and disuse atrophy [1,2].

Although different types of countermeasures, such as fitness, medication, nutrition, or special clothing, for protecting the musculoskeletal system have been developed in the last 50 years, their poor efficiency is reported in numerous studies, both during and after spaceflights [2,3,4]. A special recovery program for the musculoskeletal extensor and flexor muscles’ mass, volume, and strength decline [2,5] is required after a spaceflight due to the poor efficiency of these countermeasures.

As documented, during human exposure in real microgravity, a significant decline was reported in calf muscle performance as follows: −35% during 180 days on Mir [6] and −20 to −29% during 6 months on International Space Station (ISS). Another example was highlighted in the case of soleus muscle, with a higher atrophy, i.e., −15 ± 2%, in relation to the gastrocnemius muscle atrophy, i.e., −10 ± 2% [3]. Subjected to microgravity experiments, limb muscles present an exponential deconditioning, starting significantly even from the first days of exposure [7], where quadriceps’ strength loss reaches 11% during maximal voluntary contraction (MVC) [8] in 3-day Dry Immersion (DI) experiments.

In order to gain a better understanding on skeletal muscles disuse effects and their regenerative capacity during and after weightlessness exposure, further studies have complemented the very few data elicited from human spaceflight via different Earth-based facilities. The so-called analogues for human spaceflight and simulated weightlessness are used to induce, among other problems, musculoskeletal deconditioning affections [1,5,9]. Even though, in this regard, the most common ground-based microgravity analog is long-duration head-down tilt bed rest (HDBR) [10], the (DI) microgravity analog is much better at stimulating the space gravitational environment by the absence of support unloading [11]. Individuals are semi-flexed inside a waterproof and elastic fabric in a supine position floating via water buoyancy [10] and remaining dry. In consequence, it is observed that DI is revealing effects on the human body seven times more rapidly than HDBR [12,13]. The DI bathtubs [14] filled with water, which is automatically maintained between 32 and 34.5 °C, ensured the comfort of the subjects during the study due to the elastic, thin and large enough waterproof fabric fixed on the outside edge of the bath. The immersion bathtubs’ fabric is large enough to hold the subjects, immersed in water up to the neck, in suspension [15], a condition that mimics the “floating” of astronauts, placing less pressure on the body, for many days, used to accurately and rapidly reproduce on Earth most of physiological effects of short spaceflights.

A Dry Immersion environment induces an obvious decrease in postural muscle tone, with reduced electromyographic activity of the extensor muscles (antigravity muscles) and an increased activity of the flexor muscles within the first few hours. Subsequent studies [16] evoke an important reduction in the maximal voluntary force of antigravity muscles of the leg (m. triceps surae), with a decrease in muscle force of 19% after 7 days of DI and 4 months of HDBR, as well as a reduction in the endurance of these muscles.

Sensorimotor changes are reproduced in the first few hours and become more pronounced after 3 to 7 days of DI. These changes include perturbations in maintaining the vertical posture, disturbances in locomotor activity, and disturbances in the coordination and precision of movements. Exposure to DI triggers different disorders of postural and voluntary motor control as a result of the resetting of neuromuscular control. 

Several studies (see review by Tomilovskaya et. al [13]) concluded that the lack of gravitational support causes a reverse order in the recruitment of motor units with the suppression of small motor units and facilitation of large motor units. 

These multiple changes occurring in human motor function [17,18,19] under the conditions of real or simulated microgravity (DI, HDBR) are called hypo-gravitational motor syndrome, which includes a deficit in vestibular, proprioception, and support afferent activity and substantial alterations in the functional (atony, decline in speed-force qualities) and structural (atrophy and phenotype deterioration/domination of fast myosin heavy chain isoform expression) characteristics of skeletal muscles. 

Even though DI model studies have provided, over time, significant knowledge on microgravity-induced human physiological changes, the literature is poor in terms of DI-related microgravity effects for over 7 days. Thus, for the benefit of ultra-long space missions, the first 21-day DI campaign was undertaken that targets, among other problems, detailed musculoskeletal deconditioning experiments. The study was developed within the Russian Academy of Sciences, Russian Federation State Research Center Institute of Biomedical Problems (IBMP RAS), in 2018 [20]. Based on the 21-day DI campaign, the present paper is oriented toward the muscle-property investigations performed with the MusTone device [21,22], a myotonometric device designed and developed within The Space Applications for Human Health and Safety Department (SAHHSD), Institute of Space Science, Bucharest–Magurele, Romania. The authors hypothesized that the longer duration of the DI may affect muscle deconditioning in different patterns and also that differences between muscles’ response may occur depending on their position and role in body sustaining (deconditioning gradient/distal muscles are affected more and earlier than proximal ones).

According to Remi Demangel et al. [8], the muscle deconditioning is also associated with muscle viscoelasticity properties, which are a marker of the mechanical characteristics of the muscle. These can be evaluated with a myotonometer upon the oscillation acceleration signal. In this manner, three parameters were evaluated:

- Dynamic stiffness characterizes resistance to a contraction or to an external force that deforms its initial shape;
- Oscillation frequency is an indicator of intrinsic tension of a muscle in passive state, without voluntary contraction;
- Logarithmic decrement is a parameter that indicates muscle-elasticity capabilities and mechanical energy dissipation after deformation.

Subtle changes in muscle-viscoelasticity properties reveal the early development of muscle damage.

Muscle deconditioning involves a decrease in dynamic stiffness and oscillation frequency and an increase in logarithmic decrement.

Since the development of the MyotonPRO device developed by Myoton AS, myotonometry has become a quite often used non-invasive method in order to determine/assess mechanical properties (tone, stiffness, elasticity, relaxation, and creep) of the superficial skeletal muscles and other soft biological tissues, such as ligaments, tendons, skin, and subcutaneous tissues. The measuring method of the muscle mechanical properties with MyotonPRO consists of three main parts: (1) a short duration (15 milliseconds) with a light mechanical force (0.6 N) exertion of a mechanical impulse applied to the target muscle, (2) recording damped natural oscillation of soft biological tissue in the form of an acceleration signal [23], and (3) computation of parameters. The MyotonPRO device records information in the impact point and is able to characterize muscle health through the following parameters: oscillation frequency, logarithmic decrement, dynamic stiffness, relaxation time after mechanical stress (time required for muscle tissue to return to the shape before deformation), and ratio between muscle deformation time and relaxation time.

Compared to the MyotonPRO device, the hereby proposed MusTone [21] device can gather more information from both proximal and distal directions and can evaluate, in addition to the abovementioned parameters, others related to the propagation of the mechanical impulse along the muscle fiber, such as propagation velocity and amortization ratio. Given the presented background, the team used the updated MusTone device to measure the muscle mechanical properties in vivo in a 21-day DI experiment, as this is supposed to have more marked effects.

The mechanical wave transmission into the resting muscle mass depends on the muscle viscoelastic properties (elasticity and density) and on the contractile myofilaments and the extracellular matrix composition [24]. The team evaluated the mechanical wave characteristics by using MusTone.

## 2. Materials and Methods

### 2.1. Participants

The 21-day DI experiment on MusTone involved 6 male volunteer subjects with the right side as the dominant part, mean age 29 ± 3.68 (M ± SD) years, height 176.38 ± 4.38 (M ± SD) cm, and body weight 77.03 ± 8.97 kg (M ± SD). The anthropometric parameters of the experiment participants were within the physiological norm. The calculated body mass index (BMI) mean before the DI was 24.7 ± 2.07 kg/m2 (M ± SD). Detailed information on the subjects is given in Table 1. All the subjects signed an informed consent form to participate in the experiment, which was approved by the bioethical commission of the Institute of Biomedical Problems (IBMP) of the Russian Academy of Sciences.

Considering that the subjects investigated in our experiment are a fraction of the same batch of subjects Tomilovskaya et al. studied in Reference [25], the anthropometric parameters of normality of the subjects were checked by using the Shapiro–Wilk test, and the group was homogeneous in terms of age, weight, height, and body mass index; all of these parameters had a normal distribution (Table 1).

### 2.2. Study Procedure and Device Description

For the present 21-day DI study, the MusTone device, which is described below, was used.

**Figure 1:**
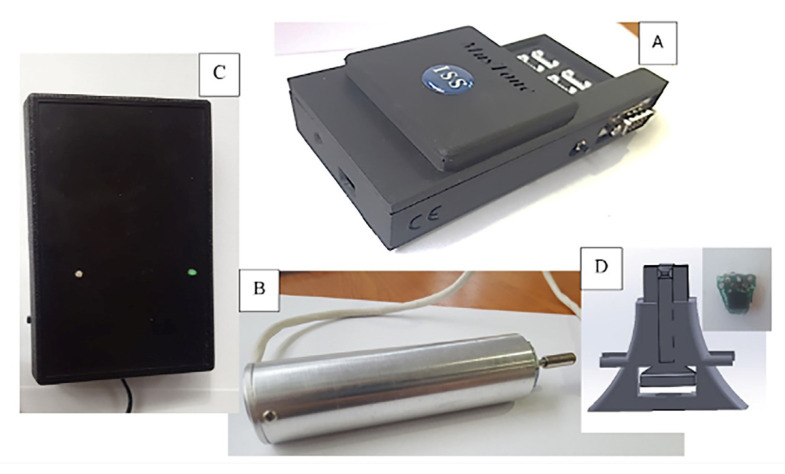
MusTone Device components: (**A**) data-processing unit, (**B**) impulse applicator, (**C**) power supply, and (**D**) accelerometers.

**Figure 2:**
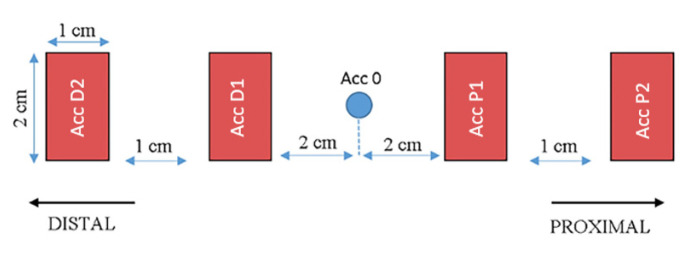
Accelerometers of interest position in relation to the impact point.

The MusTone device (Figure 1) was developed in-house within the Institute of Space Science, Bucharest, with the main purpose to record the mechanical wave propagation, using accelerometer sensors placed along and/or across the muscle fibers in order to perform complex muscular analysis. The device has great versatility, allowing us to use up to 10 accelerometers (2 on the piston and 8 placed on the skin) in the longitudinal axis of the muscle. The configuration used for this experiment consists of 2 accelerometers placed proximally (P1 and P2) and 2 accelerometers placed distally (D1 and D2) for each muscle. An analysis was performed on all the accelerometers placed on the skin closest to the impact point, both proximal and distal (P1 and D1) and at a distance (P2 and D2). The accelerometers were positioned on the skin according to the diagram presented in Figure 2.

MusTone’s functioning principle is based on a mechanical excitation applied on the tissue through a controllable percussion in regard to shape and duration, perpendicular to the direction of the muscle fiber. The acceleration signal is collected from the accelerometer associated with the striker, as well as the measurement accelerometers placed along the muscle fiber. The collected signals give information on how the mechanical perturbation propagates along the muscle fiber. 

**Figure 3:**
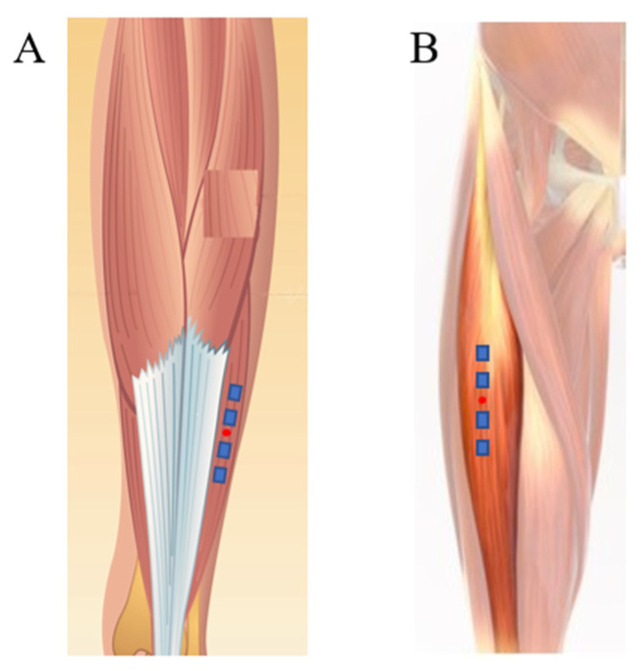
Accelerometer position on muscle bundle: (**A**) soleus [26] and (**B**) rectus femoris [27].

The muscles investigated for the present article were the soleus and rectus femoris, which are located on the right side of the body, and accelerometers were placed according to Figure 3. Considering their role in regard to body posture, the changes that occur during DI should be important. The muscle fiber and boundaries were identified by a specialist. The position of the accelerometers was marked in order to assure the experiment’s reproducibility in different experiment days.

The experiment was performed in the DI bath facility at IBMP Moscow, Russia. This particular study was without any kind of countermeasures, and inside the immersion bath, the movement was limited. The water temperature was held at 32.5 ± 2 °C. For hygiene purposes, the subjects were raised from the bath every evening for an average of 15–20 min, and most procedures were performed in the prone position of the subject. For all the experimental procedures performed during the study that required the subject to be raised from the bath for a short time, the subject was in a prone position. In their free time, they had the opportunity to read, work on a laptop, watch TV, or talk on the phone.

For the present experiment, 10 percussions were performed on each measurement, with approximately 5 s between, and the mean value was considered in order to obtain better results. The parameters used are presented in Table 2. The resulting accelerometry data were postprocessed by using MATLAB, 2010. *version R2020a*, Natick, Massachusetts: The MathWorks Inc. software, and the physiological parameters of interest were extracted.

From a statistical point of view, data were analyzed by using a comparison of the studied parameters (AR and velocity) after different intervals of dry immersion, using ANOVA mixed effects. All values were expressed as mean and standard deviation. Significance was considered against a *p*-value < 0.05.

**Figure 4:**
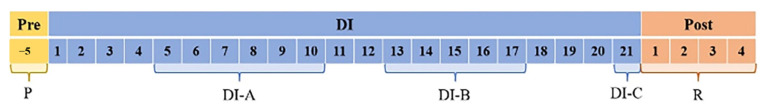
Experiment unfolding during 21-day immersion.

For the MusTone device, measurements were performed in 5 periods of time: one before the immersion, 3 measurements inside the immersion period, and 1 after the subject was extracted from the bath. The experiment unfolding is presented in Figure 4, below.

## 3. Results

The present study investigated muscles’ behavior in the longitudinal direction, parallel to muscle fascicles, using two parameters, namely the Amortization Ratio (AR) and Propagation Velocity (V); and in two directions, namely proximal (P1 and P2) and distal (D1 and D2) in different immersion days.

### 3.1. Amortization Ratio for Rectus Femoris Muscle (AR_RF)

**Figure 5:**
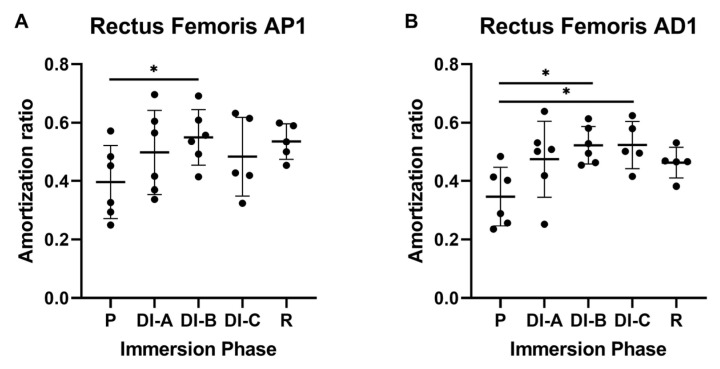
Amortization ratio for rectus femoris muscle (RF): (**A**) close proximal propagation (P1) and (**B**) close distal propagation (D1). The statistical significance of results is noted with an asterisk (*): * *p* < 0.05.

The data analysis for the rectus femoris muscle showed that, for the proximal propagation (RF_AP1), the AR increases during DI in a time-dependent manner, as seen in Figure 5A. The average value of the AR before DI was 0.396 ± 0.114 (P—preparation period). After 10 days of DI, the changes were not significant (average was 0.498 ± 0.13); instead, in DI-B, the AR value were statistically significant −0.549 ± 0.087, (*p* < 0.05).

A similar tendency was observed for the distal propagation (RF_AD1) of the mechanical impulse (Figure 5B): the average value of the AR before DI was 0.346 ± 0.091. After DI-A, the AR average became 0.475 ± 0.118, but it is still not significant (*p* > 0.05).

After prolonged DI, the AR values were statistically significant, after DI-B 0.522 ± 0.058 and DI-C 0.523 ± 0.07, *p* < 0.05, compared to the preparation period (P): 0.346 ± 0.09.

**Figure 6:**
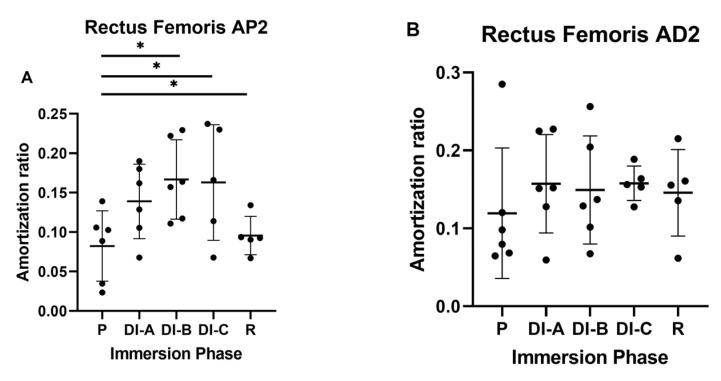
Amortization ratio for rectus femoris muscle (RF): (**A**) far proximal propagation (P2) and (**B**) far distal propagation (D2). The statistical significance of results is noted with an asterisk (*): * *p* < 0.05.

For the P2 accelerometer (RF_AP2) shown in Figure 6A, the increase of the AR was more consistent. Compared to the values collected before DI, (P = 0.082 ± 0.04), after all DI moments, the values increased significantly (*p* < 0.05): DI-A = 0.138 ± 0.047, DI-B = 0.166 ± 0.05, and DI-C = 0.162 ± 0.07.

For the D2 (Figure 6B) accelerometer (RF_AD2), no significant differences were observed between the AR values in relation to the duration of DI (*p* > 0.05). Even though the highest AR was observed for the DIB, the differences were not significant (*p* > 0.05).

### 3.2. Amortization Ratio for Soleus Muscle (AR_Sol)

**Figure 7:**
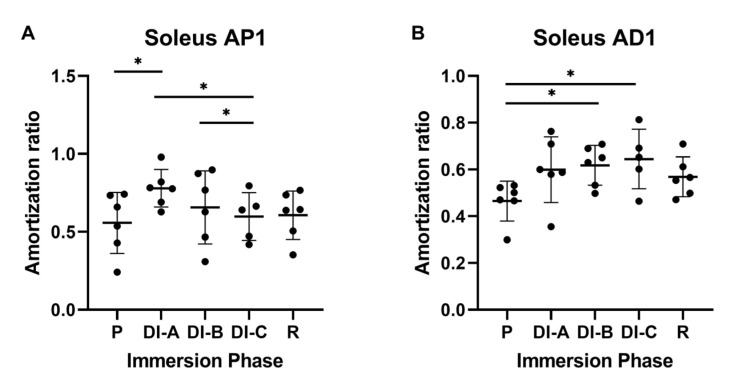
Amortization ratio for Sol: (**A**) close proximal propagation (P1) and (**B**) close distal propagation (D1). The statistical significance of results is noted with an asterisk (*): * *p* < 0.05.

The data analysis for the soleus (Sol) muscle showed a similar pattern: for the proximal propagation (Sol_AP1), the AR increased during DI, as shown in Figure 7A. The average value of the AR before DI was 0.557 ± 0.179. After DI-A, the AR average significantly increases to 0.779 ± 0.109 (*p* < 0.05). We also noted significant differences in DI-A vs. DI-C (0.779 ± 0.109 vs. 0.598 ± 0.136) and DI-B vs. DI-C (0.657 ± 0.214 vs. 0.598 ± 0.136). Regarding the distal propagation of the mechanical impulse (Sol_AD1), in Figure 7B, the average value of the AR before DI was 0.465 ± 0.077. After DI-A, the AR average increases, but it is not significant: 0.599 ± 0.128 (*p* > 0.05). After prolonged DI, the AR values became statistically significant: 0.618 ± 0.077 after DI-B, and 0.644 ± 0.113 after DI-C (*p* < 0.05).

**Figure 8:**
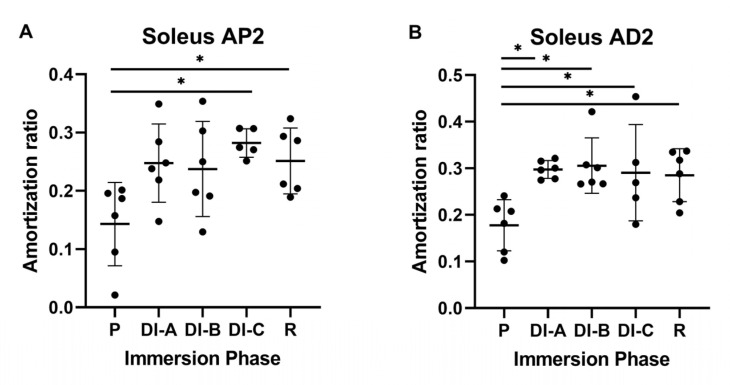
Amortization ratio for Sol: (**A**) far proximal propagation (P2) and (**B**) far distal propagation (D2). The statistical significance of the results is noted with an asterisk (*): * *p* < 0.05.

For the P2 accelerometer (Sol_AP2) shown in Figure 8A, the increase of the AR was significant after prolonged DI. Compared to P = 0.142 ± 0,07, the values increase significantly (*p* < 0.05): DI-C = 0.282 ± 0.02 and R = 0.251 ± 0.05.

For the D2 accelerometer, for all intervals of DI (DIA = 0.297 ± 0.01, DIB = 0.305 ± 0.05, DIC = 0.290 ± 0.10, and R = 0.285 ± 0.05), we observed significant increases versus P = 0.177 ± 0.05. (*p* < 0.05).

The AR is related to the muscle-structure changes that occur during prolonged DI.

Making a comparison between muscles for the P1 accelerometer on the amortization ratio, we can see that the corresponding values for Sol were significantly increased versus RF after DI-A (0.779 ± 0.109 vs. 0.498 ± 0.131) and DI-B (0.657 ± 0.214 vs. 0.55 ± 0.08), *p* < 0.05, which is consistent with the results obtained in distal direction (accelerometer D1). Meanwhile, the corresponding values for Sol were significantly increased versus RF after DI-A (0.599 ± 0.12 vs. 0.475 ± 0.118), DI-C (0.644 ± 0.113 vs. 0.523 ± 0.07 and R (0.568 ± 0.07 vs. 0.462 ± 0.04), *p* < 0.05.

In both individual muscle analyses, we observed a significantly increased AR in distal direction D1 which could express a deconditioning gradient of muscle-structure changes related to gravity absence. Both studied muscles are related to vertical posture maintenance, and the present study revealed that changes are significantly higher in the distal direction than in proximal after prolonged DI.

The comparison between muscles in the far proximal direction (P2 accelerometer) showed that the values for Sol were significantly increased (*p* < 0.05) after DI-A (0.247 ± 0.06 vs. 0.138 ± 0.04), and R (0.095 ± 0.02 vs. 0.251 ± 0.05).

A similar comparison between the Sol and RF muscles in the far distal direction (D2 accelerometer) showed significant differences after DI-A (0.297 ± 0.05 vs. 0.157 ± 0.06), DI-B (0.305 ± 0.05 vs. 0.149 ± 0.06), and R (0.284 ± 0.05 vs. 0.145 ± 0.05), *p* < 0.05.

### 3.3. Propagation Velocity (V)

**Figure 9:**
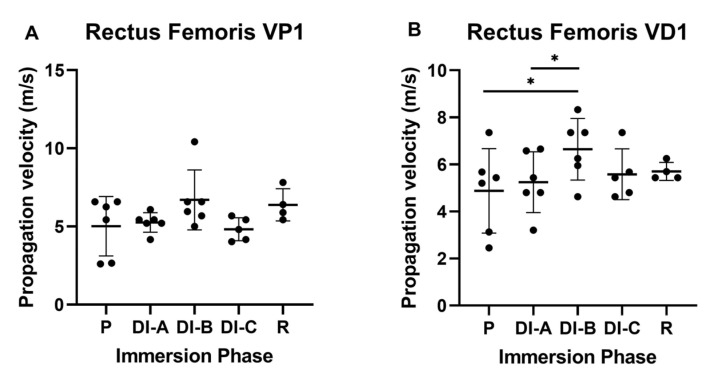
Propagation velocity for rectus femoris muscle (RF): (**A**) close proximal propagation (P1) and (**B**) close distal propagation (D1). The statistical significance of results is noted with an asterisk (*): * *p* < 0.05.

For the RF, as shown in Figure 9A, we observed a tendency of V to increase after DI-C in proximal propagation (VP1), but the values were not statistically significant. The velocity in the distal direction (VD1), as can be observed in Figure 9B, sustains the previous observation, showing a significant increase after DI-B versus P (6.645 ± 1.193 versus 4.875 ± 1.64)), *p* < 0.05. Moreover, we observed a significant increase of the same parameter in distal direction between DI-B versus DI-A (6.645 ± 1.193 versus 5.247 ± 1.179), *p* < 0.005.

For the soleus, we did not observe statistically significant differences for the V average in proximal and distal propagation.

Considering the velocity in close proximal direction for the RF and Sol muscles (P1 accelerometer), there were no observed significant differences during 21 days of DI; however, in the recovery period, a significant increase of the velocity in RF versus Sol was observed (6.386 ± 0.891 versus 5.316 ± 0.797), *p* < 0.05. The far proximal sensor, P2 accelerometer, did not record differences between the two muscles.

The same comparison in the close distal direction and far distal direction for RF and Sol did not present significant differences.

## 4. Discussion

The present study investigated rectus femoris and soleus muscle behavior in the longitudinal direction during 21 days of DI.

The literature on both human and animal experiments showed a significant decrease in muscle tone in the first days of DI, but a majority of these experiments were focused on short-term exposure, whereas, in this context, the present study offers the opportunity to analyze muscle deconditioning on long-term exposure.

Previous studies presented a large group of parameters (structural, microstructural, and functional) to characterize muscle transformation during spaceflight. For instance, the Sarcolab study [28]—performed on two astronauts after space flight on ISS—analyzed the muscles strength, size, and architecture; the cross-sectional area of the single muscle fiber; and the expression of the costameric proteins (vinculin, integrin-linked focal adhesion kinase, FAK), which were reduced in both cases, but with significant differences. There were also alterations in troponin I and troponin T in both crew members, as well as changes in actin and tropomyosin in one subject.

Several modulators of the atrophy process were also studied. In one study [29], the process of muscle hypotrophy/atrophy during spaceflight was attributed to D vitamin receptor expression, which induces myotube atrophy in vitro.

In another study [30], adiponectin alteration due to biorhythm changes was mentioned to contribute to muscle hypotrophy during space flight. The mechanisms involved the intracellular calcium content of the muscle fiber and muscle mass regulation which is why the adiponectin analogues were proposed as therapeutic solutions in spaceflight.

Changes such as the reduction in electric stimulation of the muscle fiber, the reduction in the sarcomeric proteins, and the energetic metabolism of the muscle were described [31] during hypo gravity. Moreover, changes in the myosin fibers’ phenotype in favor of the fast ones occurred.

One parameter that the team identified and analyzed is the modification of the AR parameter, and they regarded it as one of the most significant markers of muscle deconditioning; its measurement may allow for an assessment of the degree of muscle damage.

When AR was analyzed separately in the RF and Sol, respectively, we observed a significant increase of this parameter both in the proximal and distal direction, in both muscles. Significant changes appeared earlier in distal propagation than in proximal for RF, and this behavior was similar for P1 and P2 accelerometers. When comparing the AR for RF and Sol muscles, we observed that the AR increase was significantly bigger for Sol, with greater consistency in the distal direction.

In our study, prolonging the immersion process beyond 13 days demonstrated a statistically significant change for both muscles in the distal site. As the immersion period increased, the value of the AR increased. More specifically, concerning RF, an important statistical increase in the attenuation ratio was observed from DI-A to DI-C, while for the Sol muscle, this change was observed from DI-B to DI-C.

Based on these results, it may be outlined the hypothesis that muscle deconditioning occurs in a deconditioning gradient: it is possible to start first in the more distal points and to affect initially the more the distal muscles, i.e., those that are involved in posture maintenance and play an antigravity role.

This was the first time when a more complex analysis of the muscle mechanical properties was performed in a long-term DI (21 days) experiment. Four acceleration sensors (P1, P2, D1, and D2) were used to record mechanical wave propagation in proximal and distal directions in relation to the impact point.

The second analyzed parameter in our study was the propagation velocity of the mechanical wave; interestingly, for the RF, the values were significantly increased only in the distal direction, starting with DI-B. The increase of the propagation velocity can be related to a reduced muscle density, in relation to previously mentioned intracellular changes.

There were recorded significant differences between velocities of the two muscles in favor of Sol (VD2_RF versus VD2_Sol) after DI-C, in distal propagation. These data also sustain a more intense deconditioning in distal direction of the postural muscles.

The data that were obtained are consistent with previous studies [32] that used ultrasound application to induce the propagation of a mechanical shear wave. The maximum shear strain was related to the maximal force, and the reduction of this parameter was related to an increased rigidity of the muscle extracellular composition.

Considering Reference [33], it may be sustained that the long-term deconditioning after prolonged DI, expressed as the velocity propagation increase, is due to a reduced muscle density, at least partially.

One of the limitations of the present study was the small number of cases and the investigation of the muscle groups only on the right side of the body, as the target of our research did not involve any asymmetry assay.

Another limitation could be the investigation of only longitudinal direction muscle behavior; however, as stated in Reference [34], muscle deconditioning involves mainly a decrease in strength that is more important than the reduction in muscle mass or volume.

Further studies prove that gravity absence creates not only anatomical, biochemical, and functional changes in the muscles but also acts in relation to time and the muscle position/role in the human body. Considering the extensive DI experiment and the large amount of data collected by different participants on a wide range of devices, a joint article for a comparison with a well-known device is being taken into consideration.

## 5. Conclusions

Muscle deconditioning is time dependent; as the exposure of muscle to DI increases, the AR increases.

The changes of the AR are more significant in both muscles in the distal direction. Basically, it can be postulated that the lower fibers would be most affected probably due to the lack of the pressure to which the fibers are subjected in an orthostatic position in normal conditions. Prolonging the immersion process by more than 10 days demonstrates a statistically significant change for both studied muscles, especially for the accelerometers positioned at the distal level.

Based on these results, one may sustain the hypothesis that muscle deconditioning occurs in an orthostatic gradient: it starts first in distal points and more greatly affects the distal muscles, those that are involved in posture maintenance and which have an essential antigravity role. From what we know, this was the first time a muscle-deconditioning gradient was detected and described. This was possible due to the special features of the MusTone device that allowed us to collect data simultaneously in many points along the muscle fiber. Further studies will be necessary to consolidate our results.

Due to the development of space tourism, astronauts and space tourism candidates will require a fast, non-invasive, reliable method that can be used to rapidly quantify the muscle dynamic changes in a real-time manner.

## Figures and Tables

**Table 1:** Subject’s body parameters.

	Age	Height	Body Mass	Index of Body Mass
S1	23	174	81.7	27.0
S2	30	183	86.9	25.9
S3	32	180.1	80.8	24.9
S4	26	176.5	81	26.0
S5	32	172	64.2	21.7
S6	31	172.7	67.6	22.7
Mean value	29	176,383	77,033	24,700
SD	3.688	4.383	8.975	2.072
SEM	1.506	1.789	3.664	0.846

**Table 2:** Parameters used for the present DI experiment.

Parameter	Set Value	Measurement Unit	Observations
Acquisition step	0.2	ms	Values are recorded each 0.2 ms
Stimulus action time	10	ms	Time necessary for the percussion to be applied
Acquisition start	−5	ms	If the value is negative, the acquisition starts before the impulse is applied
Acquisition time	650	ms	The duration of the recording
Scale	±4 g	m/s2	Amplitude scale

## Data Availability

The data presented in this study are available upon request from the corresponding author. The data are not publicly available due to the contract signed between institutions participating in the experiment.
